# Fifteen Years Controlling Unwanted Thoughts: A Systematic Review of the Thought Control Ability Questionnaire (TCAQ)

**DOI:** 10.3389/fpsyg.2019.01446

**Published:** 2019-06-19

**Authors:** Albert Feliu-Soler, Adrián Pérez-Aranda, Jesús Montero-Marín, Paola Herrera-Mercadal, Laura Andrés-Rodríguez, Natalia Angarita-Osorio, Alishia D. Williams, Juan V. Luciano

**Affiliations:** ^1^Institut de Recerca Sant Joan de Déu, Esplugues de Llobregat, Spain; ^2^Teaching, Research and Innovation Unit-Parc Sanitari Sant Joan de Déu, St. Boi de Llobregat, Spain; ^3^Primary Care Prevention and Health Promotion Research Network (RedIAPP), Madrid, Spain; ^4^Dharamsala Institute of Mindfulness and Psychotherapy, Zaragoza, Spain; ^5^Aragon Institute for Health Research (IIS Aragon), Zaragoza, Spain; ^6^Department of Psychology, Faculty of Science, University of New South Wales, Sydney, NSW, Australia

**Keywords:** systematic review, TCAQ, thought control, thought suppression, reliability, validity, quality assessment

## Abstract

Thought control ability is a vulnerability factor implicated in the etiology and maintenance of emotional disorders. This manuscript aims to systematically review the use and psychometric performance of the Thought Control Ability Questionnaire (TCAQ), designed to assess people's ability to control unwanted thoughts. Three electronic databases were searched for papers administering the TCAQ published in indexed peer-reviewed journals. Data (participants characteristics, country, study design, etc.) were extracted from the results for qualitative synthesis. The TCAQ's content validity, dimensionality, internal consistency, test-retest reliability, convergent/divergent validity, floor/ceiling effects, and interpretability were summarized. Two reviewers independently screened articles and assessed quality taking COSMIN criteria into account. Finally, the review included 17 papers. The TCAQ has been administered to healthy individuals, students, and adult patients, in six languages from nine countries. We found that the TCAQ, and its shorter versions, demonstrate robust reliability and adequate content validity. Of interest is the TCAQ's capacity to predict performance in diverse experimental tasks focused on thought control. The TCAQ unidimensionality has been supported in exploratory and confirmatory factor analyses. Regarding construct validity, the TCAQ is significantly related to a wide range of psychopathological measures of anxiety, worry, depression, obsessive-compulsive symptoms, etc. However, as only a few of the included studies had a longitudinal design, we are unable to draw firm conclusions about the measure's temporal stability. Moreover, psychometric aspects such as factorial invariance across different samples have not been analyzed. Despite these limitations, based on available psychometric evidence we can recommend using the TCAQ for measuring perceived control of unwanted thoughts.

## Introduction

Individuals can experience unwanted thoughts about events that have occurred in the past (e.g., the death of a loved one), that might happen in the future (e.g., end of employment contract), or that may never happen at all (e.g., alien invasion). The unwanted thoughts experienced by healthy individuals are similar in form and content to the thoughts reported by patients suffering from psychopathology such as major depression, obsessive-compulsive disorder, or posttraumatic stress disorder. However, healthy subjects and patients do differ with respect to the frequency, intensity, disturbance, etc., elicited by these unwanted thoughts (Clark and Rhyno, 2005).

Perceived control over such unwanted thoughts, and the repertoire of associated cognitive and behavioral responses activated in efforts to control such thoughts, may distinguish benign thoughts from those that become pathological. Terms such as “perceived control,” “thought control ability,” and “thought suppression,” have been used interchangeably to define a set of highly related constructs. Notwithstanding, there are subtle conceptual differences among them. In general, “perceived control” has been defined as a personal belief about one's capacity to control internal emotional reactions to threats or external events (Mardiyono et al., 2011), whereas “perceived thought control ability” (a construct equivalent to “thought control self-efficacy” coined by Bandura, 1997) is the perceived capacity to self-regulate thought processes, a construct that plays an important role in the maintenance of well-being. “Thought suppression” was defined by Wegner and Zanakos (1994) as the general tendency or desire to suppress distressing thoughts.

The topic of thought control has generated a great body of work in the fields of clinical and experimental psychology in the last two decades, with research indicating both beneficial (Engen and Anderson, 2018) and counterproductive effects (Magee et al., 2012). For example, the seminal work of Wegner (1994) underscored the detrimental effects of thought suppression (the white bear effect), whereas recent research indicates that “failures in suppression” (rather than thought suppression) predict psychopathology (Engen and Anderson, 2018; Hulbert and Anderson, 2018). Like Bandura (1997), these authors posit that perceived thought control has a positive role in mental well-being. In line with this, contemporary theories claim that low perceived thought control is strongly related to the experience of negative emotions and might be considered a general psychological vulnerability factor in the etiology and maintenance of emotional disorders (Gallagher et al., 2014).

### The Assessment of Thought Control

Various self-report measures have been developed in order to assess individual differences in thought suppression and thought control, as outlined below (see Table 1).

**Table 1:** Outline of thought control/suppression measures.

**References; Country**	**Measure**	**Items**	**Description**
Wells and Davies (1994); UK	TCQ	30	The TCQ is a self-report questionnaire that assesses the frequency of use of five strategies to coping with unwanted thoughts (distraction, social control, punishment, worry, and reappraisal). Each item is answered on a 4-point scale (1, never; 2, sometimes; 3, often; and 4, almost always)
Wegner and Zanakos (1994); USA	WBSI	15	Self-report inventory that assesses people's general tendency to suppress unwanted thoughts. Answers are given on a five-point Likert scale ranging from A (strongly disagree) to E (strongly agree).
Rassin (2003) van Schie et al. (2016); Netherlands	TSI/TSI-R	15/21	Self-report inventory to measure successful and unsuccessful thought suppression. Items are scored from 1 “strongly disagree” to 5 “strongly agree.” The TSI-R consists of 21 items.
Luciano et al. (2005); Spain	TCAQ	25	Self-report questionnaire to assess individual differences in the perceived ability to control unwanted intrusive thoughts (“*I often cannot avoid having upsetting thoughts*”). Items are rated on a 5-point Likert-type scale (A = strongly disagree, E = strongly agree). See item content in Supplementary Table 1.

*TCAQ, Thought Control Ability Questionnaire; TCQ, Thought Control Questionnaire; TSI, Thought Suppression Inventory (– Revised); WBSI, White Bear Suppression Inventory*.

#### Thought Control Questionnaire (TCQ)

Wells and Davies (1994) developed this 30-item measure to assess the specific strategies people use to control their unwanted thoughts. Originally, these strategies were grouped into five empirically derived subscales: “distraction” (e.g., *I call to mind positive images instead*), “social control” (e.g., *I find out how my friends deal with these thoughts*), “worry” (e.g., *I dwell on other worries*), “punishment” (e.g., *I punish myself for thinking the thought*), and “reappraisal” (e.g., *I try a different way of thinking about it*). The dimensionality, reliability and validity of the TCQ was evaluated in 108 students (study 1) and 208 patients with different anxiety disorders (study 2) by Fehm and Hoyer (2004), who found that the five thought control strategies were related to measures of psychopathology in both samples, with “punishment” and “worry” considered maladaptive. However, the dimensionality was questioned because the exploratory factor analyses (EFA) revealed that some items produced non-significant loadings on their respective factors or significant loadings on the factors they were not assigned to in the original version. Similarly, Luciano et al. (2006) computed a confirmatory factor analysis (CFA) with the purpose of analyzing the goodness-of-fit of the five-factor model initially proposed by Wells and Davies (1994) and found that many items did not load on their respective factor, suggesting the need for item refinement.

#### White Bear Suppression Inventory (WBSI)

Wegner and Zanakos (1994) developed this 15-item self-report measure to assess people's dispositional tendency to suppress thoughts. Subsequent studies indicated that the WBSI does not capture only one factor, but at least one other construct called “intrusions” (e.g., Blumberg, 2000; Höping and de Jong-Meyer, 2003; Rassin, 2003; Luciano et al., 2006; Schmidt et al., 2009), which indirectly refers to difficulties in the control over unwanted thoughts. The “intrusions” factor showed moderate correlations with measures of anxiety and depression, whereas the “thought suppression” factor was not associated with these psychopathological indicators. For this reason, Höping and de Jong-Meyer (2003) highlighted the need of differentiating between the perceived ability to suppress and the tendency to suppress, when trying to establish a link between thought control and psychopathology. This controversial debate on the structure of the WBSI triggered the development of two other self-reports: the *Thought Suppression Inventory* (TSI; Rassin, 2003) and the *Thought Control Ability Questionnaire* (TCAQ; Luciano et al., 2005). Both attempted to overcome the WBSI dimensional shortcomings by computing separate scores for three different constructs (TSI; intrusions, suppression attempts, and successful suppression) or by generating an item set that entirely focused on the assessment of perceived ability to control unwanted thoughts (TCAQ).

#### Thought Suppression Inventory (TSI)

The TSI comprises 15 items distributed into three 5-item subscales measuring “intrusion,” “suppression attempts” and “successful/effective thought suppression” dimensions. Rassin (2003) reported adequate internal consistency, test-retest reliability, and convergent validity of the TSI in two student samples. Although Wismeijer (2012) confirmed the original 3-factor model proposed by Rassin (2003), he suggested that eight items should at least be rephrased or even removed after using CFA and Mokken scale analysis. More recently, van Schie et al. (2016) developed the TSI-R. These authors rephrased or replaced the problematic items of the TSI and some new items were added. The TSI-R items were analyzed in a sample of Dutch respondents from different age groups. The hypothesized three-factor model was well-represented in the youngest age group. In the middle and old age groups the “intrusion” and “successful/effective thought suppression” scales seemed to be sound scales, but the “thought suppression attempts” scale for these age groups was found to be problematic. Further research with the TSI-R in non-Dutch clinical and non-clinical samples seems necessary because to our knowledge, the instrument has not been tested in other cultures or languages.

#### Thought Control Ability Questionnaire

(TCAQ; Luciano et al., 2005; see Supplementary Table 1). The TCAQ is a 25-item self-report measure that was designed to assess individual differences in the perceived ability to control unwanted thoughts (see Supplementary Material). The original study (Luciano et al., 2005) was conducted with undergraduate students, and the authors reported that the measure was unidimensional, had excellent internal consistency (α = 0.92), adequate test-retest reliability (*r* = 0.88) and convergent validity even after controlling for other thought control measures (TCQ and WBSI). Since its publication in 2005, the TCAQ has been requested widely within the clinical psychology field, as well as outside this discipline, for use in many different types of studies carried out in diverse cultures.

### Objective of the Systematic Review

Given that the TCAQ has been available for more than a decade up to this point, with more than 30 references to Luciano et al. (2005) in the ISI Web of Knowledge (2018), it is time for a systematic review of its worldwide use, and analyses of strengths and weaknesses in diverse contexts and cultures. To date, there are no previous reviews synthesizing available information on the TCAQ, as has been done in similar instruments such as the WBSI (Schmidt et al., 2009). The present systematic review bridges the aforementioned gap by summarizing the available data on the use of the TCAQ and by critically appraising its psychometric properties, taking Terwee et al. (2007) quality criteria as a guiding framework.

## Methods

The review protocol was registered in the PROSPERO electronic database of prospectively registered systematic reviews in health and social care on October 20th, 2017 (registration number: CRD42017080218). We report this systematic review following the Preferred Reporting Items for Systematic Reviews and Meta-analyses guidelines (PRISMA, http://www.prisma-statement.org/, Moher et al., 2009). Given that the TCAQ is not a patient-reported outcome measure, we followed the recommended Consensus-based Standards for the selection of health Measurement Instruments (COSMIN) guideline for systematic reviews as much as possible (Prinsen et al., 2018). COSMIN checklist is a tool designed specifically for systematic reviews on psychometric studies (https://www.cosmin.nl/).

### Search Strategy

We performed the literature search in the following electronic bibliographical databases: PubMed, PsychINFO, and EMBASE. We searched manuscripts published in peer-reviewed journals at any time from 2005 (year the TCAQ was published) until October 10th 2017 (search updated in October 10th 2018). We used the following search terms in all fields: “Thought Control Ability Questionnaire” OR “TCAQ,” without using limitations *a priori* (e.g., “humans” or “English” language). There were no restrictions related to age. Additionally, we also searched Google Scholar to ensure we did not miss potentially relevant articles.

### Eligibility Criteria

We considered journal articles for which the abstract and full text were published in English or Spanish, and used the TCAQ or its brief versions as outcomes. Literature reviews, books, dissertations, commentaries, conference abstracts, study protocols, case-reports, qualitative studies, non-peer-reviewed manuscripts, and non-English or non-Spanish papers were excluded. We excluded unpublished dissertations, master's theses, or conference presentations because restricting our analyses to studies published in peer-reviewed journals increased the likelihood that studies would be of at least minimal acceptable quality and would be relatively accessible.

### Study Selection

The search hits were inserted in citation files (using EndNote software) and duplicates were removed (Stage 1). The titles and abstracts were separately screened for eligibility by two co-authors (Stage 2: A.P-A and J.V.L). The full text of all potentially relevant manuscripts (where the abstract did not provide enough details to confirm eligibility) were downloaded, examined, and discarded from the systematic review if they did not meet the inclusion criteria (Stage 3). References of the included papers were also examined in depth and cross-checked (Stage 4). Data extraction of included papers was made by two independent researchers (A.P-A and J.V.L) using a standardized extraction sheet form with any disagreements resolved through discussion (Stage 5). Disagreements not resolved by the two co-authors were arbitrated by a third co-author (A.F-S). Information was extracted on: Year of publication; Study design (i.e., psychometric study, experimental study etc.); Country where the study was conducted; Sample size; Sample type (i.e., general population, clinical sample, etc.); TCAQ version used (i.e., TCAQ-25, TCAQ-23, or TCAQ-20).

### Inter-Rater Agreement

We computed inter-rater agreement for final inclusion/exclusion of the studies between the two evaluators using kappa (κ), which is a chance corrected measure of inter-rater reliability, and ranges from −1 to +1, with +1 being perfect agreement, −1 being perfect disagreement, and 0 being agreement no better than chance. Agreement is graded as follows: poor (0.00), slight (0.01–0.20), fair (0.21–0.40), moderate (0.41–0.60), substantial (0.61–0.80), and almost perfect (0.81–1.00).

### Quality Assessment

The psychometric properties of the TCAQ were reviewed and rated for quality based mainly on Terwee et al. (2007) quality criteria for health status measures. Considering these criteria were developed for measures of health status, Barker et al. (2002) “rules of thumb” for evaluating psychological tools were also considered. The following psychometric domains were evaluated: (1) content validity, (2) factor structure, (3) internal consistency, (4) test-retest reliability, (5) convergent and discriminant validity, (6) floor and ceiling effects, and (7) interpretability. As per Strauss et al. (2016), we did not include criterion validity or responsiveness as quality criteria given the nature of the TCAQ. Following Strauss et al. (2016) procedure, the TCAQ received a score of 2 if there was evidence for the specific criterion being fully met, 1 if the criterion was partially met, and 0 if the criterion was not met, or if no relevant data were reported. Scores in the seven psychometric domains were summed to provide an overall quality rating. Therefore, the total quality score for the TCAQ could range between 0 and 14.

1. *Content validity* (i.e., the extent to which the construct of interest is comprehensively captured by the items in the TCAQ). Under this criterion, Terwee et al. (2007) highlight the importance of defining the measurement aim of the questionnaire, the target population for which the questionnaire was developed, with both individuals of the target population and experts being involved in item development. For this criterion to be fully met (2 points), TCAQ items must have been designed in consultation with individuals from the target population and experts on thought control.
2. *Factor structure* (i.e., analysis of the dimensionality of the measure has been examined and supported). This criterion was added by Strauss et al. (2016). A score of 2 was given if exploratory factor analysis (EFA) and confirmatory factor analysis (CFA) had been carried out in different samples or if a CFA had been computed taking a theoretical model into account (a score of 2 was only given if the factor analyses support the proposed one-factor structure). Rules-of-thumb in factor analysis vary from 5 to 10 respondents per item, with a minimum number of 100 subjects to ensure stability of the variance-covariance matrix. A score of 1 was given if only EFA has been conducted (without CFA) and if the EFA supports the factor structure. A score of 0 was given where either factor analysis has not been conducted or where EFA and/or CFA have been conducted and results do not support the proposed unidimensionality.
3. *Internal consistency* (i.e., the extent to which TCAQ items are inter-related and measure the same construct). According to Terwee et al. (2007) criteria, Cronbach's α had to be between 0.70 and 0.95.
4. *Test–retest reliability* (i.e., stability of the measured construct over time). According to Barker et al. (2002) “rules of thumb” the minimum test–retest reliability had to be *r* = 0.70 for this criterion to be fully met. Although no consensus about the required time period between administrations currently exists, 1 or 2 weeks is considered sufficient to prevent recall. The intra-class correlation coefficient (ICC) is the most recommended reliability statistic for continuous measures.
5. *Convergent and discriminant validity* (i.e., the extent to which scores on the TCAQ are significantly associated with other theoretically related measures). For this criterion to be met, Terwee et al. (2007) require that (i) specific hypotheses are formulated by the authors about expected correlations and (ii) at least three quarters of results are in line with expectations. Following Barker et al. (2002), at least two correlations with theoretically related constructs had to be least *r* = 0.50 to support convergent validity.
6. *Floor and ceiling effects* (i.e. number of respondents achieving the highest (25) or lowest possible (0) scores). No more than 15% of the sample should obtain the top or bottom score on the TCAQ to meet this criterion (McHorney and Tarlov, 1995).
7. *Interpretability* (i.e., how differences in scores on the TCAQ can be interpreted, or the degree to which qualitative meaning can be obtained from quantitative scores). Terwee et al. (2007) suggest that means and SDs of scores from at least four relevant subgroups of participants to be reported (e.g., TCAQ scores in males vs. females, healthy subjects vs. patients, etc.), that is, a known-groups validity approach is suggested with means and SDs of scores of relevant subgroups of subjects who are expected to differ in the TCAQ.
8. Two co-authors (A.P-A and JVL) independently scored the TCAQ employing these criteria, and discrepancies were resolved by discussion.

## Results

### Selection and Inclusion of Studies

**Figure 1:**
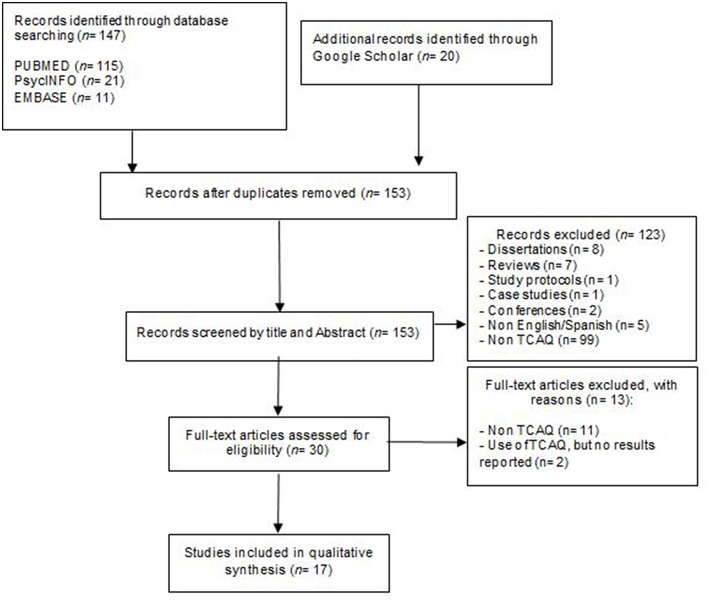
PRISMA flow diagram from record identification to study inclusion.

The systematic search identified 167 journal articles (153 abstracts in total after removal of duplicates). One-hundred and twenty-three articles were excluded in the process of title and abstract review. Thirty potentially eligible full-text papers were examined in detail for further consideration, and 13 of these were excluded. Finally, 17 studies met the inclusion criteria. Additional references were sought from these manuscripts reference lists, but this did not yield new references meeting the inclusion criteria. There was an “almost perfect” inter-rater agreement between the two raters (kappa: κ = 0.89). See Figure 1 for a flowchart of the process.

### Characteristics of Included Studies

A detailed description of the information and data extracted from the 17 reviewed manuscripts is presented in Table 2. The included studies were carried out in nine different countries: the UK (*n* = 2), the Netherlands (*n* = 3), Switzerland (*n* = 3), Spain (*n* = 2), USA (*n* = 2), Australia (*n* = 2), Brazil (*n* = 1), Cuba (*n* = 1), and China (*n* = 1).

**Table 2:** Descriptive data of the selected articles (*n* = 17) in chronological order.

**References; Country**	**TCAQ version/Language**	**Study design**	**Target population**	**Sample size**	**Age (years M + SD); Gender (% female)**	**Results about TCAQ**
Luciano et al. (2005); Spain	TCAQ-25 Spanish	Psychometric study	Undergraduate students	211	21.4 (4.8) 83.9%	•Mode of TCAQ administration: computer. •Initial pool of 42 items; 17 items were eliminated due to low corrected item-total correlations. •TCAQ scores: T1 83.6 (*SD* = 17) and T2 84.8 (*SD* = 17) •PCA (Varimax rotation): One component that explained 35.4% of the variance. All factor loadings >0.45 •α = 0.92; test–retest reliability (2-months) = 0.88 (Pearson correlation) •Validity: significant correlations with trait anxiety (STAI-T, *r* = −0.82), worry (PSWQ, *r* = −0.74), neuroticism (EPQ-N, *r* = −0.72), depression (BDI-I, *r* = −0.68), obsessive-compulsive symptoms (MOCI, *r* = −0.50), thought suppression (WBSI, *r* = −0.69), guilt feelings (SC-35, *r* = −0.67), and tendency to use thought control strategies (TCQ, *r* = −0.23). The TCAQ predicted depressive symptoms (BDI-I), obsessive–compulsive complaints (MOCI), pathological worry (PSWQ) and guilt feelings (SC-35) after controlling for WBSI and TCQ.
Luciano and Algarabel (2006); Spain	TCAQ-25 Spanish	Correlational study	Undergraduate students	63	22.0 (6.5) 77.8%	•Mode of TCAQ administration: computer. •Validity: repressors (high anxiety measured with the STAI-T and high defensiveness measured with the MCSDS) and low anxious individuals scored significantly higher on the TCAQ than did high anxious and defensive high anxious individuals. Level of perceived thought control ability depends on trait anxiety, rather than the combination of trait anxiety, and defensiveness.
Gay et al. (2008); Switzerland	TCAQ-25 (Study 1) French TCAQ-23 (Study 2) French	Psychometric study	Undergraduate students	Study 1 (207) Study 2 (254)	23.2 (5.3) 71.5% 22.7 (5.4) 74.0%	Study 1 •TCAQ scores: 76.98 (*SD* = 14.97) •Intraclass correlation = 0.28, 95% CI 0.24–0.32 •EFA with one factor that explained 29.6% of the variance •Item removal: Items 5 and 8 had small loadings (<0.30) and capture behavior control. Study 2 •TCAQ scores: 68.31 (*SD* = 16.27) •Intraclass correlation = 0.38, 95% CI 0.34, −0.43 •EFA with one factor explained 39.4% of the variance and all loadings were close to or >0.40. •The ICC (C,K; equivalent to α) = 0.93, 95% CI 0.92, 0.95 •CFA: RMSEA = 0.085, 90% CI = (0.078, 0.093) and SRMR = 0.062. All standardized loadings were >0.40 •Men had higher TCAQ scores, r_point−biserial_ = 0.29, 95% CI 0.17–0.39, as did older participants *r* = 0.13, 95% CI 0.01–0.25. •Validity: significant correlations with worry (PSWQ, *r* = −0.82) and obsessive-compulsive symptoms (OCI-R subscales, *r* ranging from −0.24 to −0.79).
Peterson et al. (2009); USA	TCAQ-25 English	Correlational study	Undergraduate students	283	N.A 82.3%	•Mode of TCAQ administration: computer. •α = 0.89 (male), 0.91 (female) •TCAQ scores: male participants (*M* = 82.43, *SD* = 13.97) > female participants (*M* = 73.54, *SD* = 15.01)
						•Validity: In male participants, TCAQ correlated with 5 of the 11 main clinical syndrome scales (ANX, DEP, PAR, SCZ, and BOR, r ranging from−0.43 to−0.80) and with 3 of 4 treatment consideration scales (SUI, NON, and RXR, r ranging from−0.46 to−0.60). In female participants, the TCAQ correlated with 8 of the 11 main clinical syndrome scales (SOM, ANX, ARD, DEP, MAN, PAR, SCZ, and BOR, r ranging from−0.19 to−0.73) and all 4 treatment consideration scales (SUI, STR, NON, and RXR, r ranging from−0.34 to−0.53). Multiple regression analyses by gender confirmed these relationships (controlling for perceived stress).
Grisham and Williams (2009); Australia	TCAQ-25 English	Correlational and Experimental study	Undergraduate students	166	19.77 (3.64) 68.7%	•α = 0.89 •Validity: The TCAQ was negatively associated with the OCI-R (-0.52), BDI-II (-0.66), BAI (-0.51), RRS-RSQ Brooding (-0.67), RRS-RSQ Reflection (-0.31), and RRS-RSQ Depression (-0.69)•In the standard thought suppression task, the TCAQ significantly predicted frequency of the target thought (accident of a loved one) during the experimental period. TCAQ scores approached significance in predicting unique variance in distress (controlling for BDI-II and BAI).
Williams et al. (2010); Australia	TCAQ-25 TCAQ-20 (Study 1) English TCAQ-20 (Study 2) English	Study 1 Psychometric study Study 2 Experimental study	Undergraduate students	Study 1 (720) Study 2 (71)	19.5 (3.3) 67.2% 19.6 (2.2) 67.6%	Study 1 •CFA TCAQ-25: RMSEA = 0.077 and SRMR = 0.071. Five items with standardized loadings <0.40 (Items 5, 7, 8, 14, and 25). •CFA TCAQ-20: RMSEA = 0.079 and SRMR = 0.061. All standardized loadings >0.40. TCAQ-20 retained for the remaining analyses. •TCAQ scores: male participants (*M* = 59.83, *SD* = 13.497) > female participants (*M* = 57.27, *SD* = 13.30) •α = 0.88; test-retest reliability at 6 months *r* = 0.68 (Pearson correlation). •Validity: Negative correlation with BDI-II (*r* = −0.35), BAI (*r* = −0.48), DASS-21 (r = −0.27), OCI-R (r = −0.51), and maladaptive cognitive control strategies (TCQ-worry and TCQ-punishment, *r* = −0.12, and WBSI, *r* = −0.47). Study 2 •α = 0.91 •Validity: In the standard thought suppression task, TCAQ scores explained significant variance in frequency, distress ratings, and suppression efforts during the suppression periods. Weak perceived thought control ability (low TCAQ scores) predicted higher frequency and associated distress of a self-relevant target thought and higher suppression efforts (controlling for DASS and OCI-R scores).
Gay et al. (2011); Switzerland	TCAQ-23 French	Correlational study	Undergraduate students	Study 1 (250) Study 2 (97)	23.5 (5.8) 67.2% 21.4 (4.3) 100%	Study 1 •α = 0.91 •Validity: Thought control ability (TCAQ scores) were negatively related to two facets of impulsivity (urgency, *r* = −0.53, and lack of perseverance, *r* = −0.20) measured with the UPPS in the whole sample.
						Study 2 •α = 0.92 •Validity: Thought control ability (TCAQ scores) were negatively related to two facets of impulsivity (negative urgency, *r* = −0.54 and lack of perseverance, *r* = −0.34) measured with the UPPS in the whole sample, the WBSI (*r* = −0.64 intrusions and thought suppression subscales), pathological worry (PSWQ, *r* = −0.70), and the obsessing subscale of the OCI-R (*r* = −0.70). The regression analyses indicated that among the measures assessing intrusive thoughts (TCAQ, WBSI, OCI-R-Obsessing, PSWQ), the TCAQ showed the highest percentage of variance that could be explained by impulsivity.
Gootjes et al. (2011); Netherlands	TCAQ-25 Dutch	Experimental study	Healthy adults (Yogic vs. Non-Yogic practitioners)	24	Yogic group (*n* = 12) 23.1 (2.6) Non-yogic group (*n* = 12) 22.6 (2.7) Gender N.A	•Validity: The Yogic practitioners tended to score higher on the TCAQ (*M* = 89.8 *SD* = 10.8) compared to controls (*M* = 83.5 = 10.3), but this difference did not reach statistical significance (lack of statistical power).
Valdez and Lilly (2012); USA	TCAQ-20 English	Correlational study	Undergraduate students that are interpersonal trauma (IPT) survivors	171	19.7 (2.5) 66.7%	•Mode of TCAQ administration: computer. •α = 0.92 •Validity: More IPT experiences (TLEQ) were associated with less perceived ability to control unwanted, intrusive thoughts. Weak thought control ability was also associated with increased post-traumatic stress symptoms (SCL-PTSD, *r* = −0.70). Finally, weak thought control ability was a significant mediator of the relationship between multiple IPT victimizations and greater PTSS. A multiple mediation model revealed that TCQ-punishment and WBSI-suppression were unique mediators of the relation between TCAQ scores and PTSS severity.
Küpper et al. (2014); UK	TCAQ-25 English	Experimental study	Healthy adults	24	22.3 (N.A) 75%	•Validity: Participants were split into two groups with higher and lower self-rated mental control abilities according to the TCAQ. In the think/no-think task (TNT), high-control participants showed more forgetting (inhibitory control) than did low-control participants and showed more suppression of event details. Similarly, there were significant negative correlations between TCAQ scores and “no-think” recall. Thus, voluntary forgetting during an experimental task is linked to people's perceptions of their thought control capacity in daily life.
Gootjes and Rassin (2014); Netherlands	TCAQ-25 Dutch	Correlational study	Healthy adults	104	43.7 (11.4) 70.2%	•Validity: More time spent meditating and more mindfulness (MAAS) are both associated with greater perceived thought control ability (TCAQ, *r* = 0.51). Higher perceived TCAQ scores were associated with greater level of dispositional optimism (LOT, *r* = 0.73), greater level of perceived social connectedness (SCS-R, *r* = 0.52), more positive affect (PANAS-PA, *r* = 0.46), less trait anxiety (STAI-T, *r* = −0.83) and less negative affect (PANAS-NA, *r* = −0.71).
Rodríguez-Martín et al. (2015); Cuba	TCAQ-7 Spanish	Correlational study	Healthy adults	1,184	32.9 (12.9) 69.1%	•α = 0.83 •Validity: Overweight/Obese individuals scored significantly lower on thought control ability (TCAQ) than normal-weight participants.
Catarino et al. (2015); UK	TCAQ-25 English	Experimental study	Adults with PTSD Trauma-exposed adults without PTSD	18 18	34 (13) 61.1% 37 (14) 61.1%	•Validity: TCAQ scores of PTSD participants (*M* = 55.7, *SD* = 14.7) <non-PTSD participants (*M* = 83.9, *SD* = 14.6). In the TNT task (all participants), suppression-induced forgetting of details and TCAQ scores had a robust positive correlation (it remained significant even after controlling for BDI-II scores).
van Schie et al. (2016); Netherlands	TCAQ-25 Dutch	Psychometric study	Healthy adults stratified by age	351 ( ≤ 25 years) 202 (26-50 years) 231 (≥ 51 years)	20.2 (2.19), 71.5% 36.1 (7.79), 66.3% 62.4 (7.61), 57.1%	•Reliability λ2 = 0.92 (≤25 years);0.93 (26–50 years);0.92 (≥51 years) •Validity: Thought control ability (TCAQ scores) were negatively related with the dimensions “intrusion” (*r* ranging from −0.72 to −0.78) and “suppression” (*r* ranging from −0.13 to −0.25) and positively correlated with “effective suppression” (*r* ranging from 0.62 to 0.65), measured with the TSI-R in the stratified samples.
Piguet et al. (2016); Switzerland	TCAQ-23 French	Experimental study	Patients with major depression or bipolar disorder Healthy adults	29 32	N.A.	•Validity: TCAQ scores of patients (*M* = 58.7, *SD* = 13.3) < healthy participants (*M* = 79.3, *SD* = 15.9). The TCAQ did not explain between-group differences in the task-switching paradigm (used for the generation and inhibition of mental sets).
Lu et al. (2017); China	TCAQ-25 Chinese	Correlational study	Undergraduate Students Patients with major depression	Study 1 (74)Study 2 (148)Study 3 (21 MDD)	22.2 (1.1) 50.0% 19.5 (1.1) 75.7% 31.1 (9.5) 76.2%	Study 1 α =0.89 •Validity: The TCAQ presented significant correlations with neuroticism (NEOPI-R, r = −0.36), rumination (SRRS, r = −0.24), and depressive symptoms (BDI-II, r = −0.43) among healthy subjects. SRRS (but not TCAQ) had a mediating role between neuroticism (NEOPI-R) and depressive symptoms (BDI-II). Study 2 •α = 0.89 •Validity: Replication of mediation model with life event stressors (ASLEC) as covariate. Both SRRS and TCAQ had a mediating role between neuroticism (NEOPI-R) and depressive symptoms (BDI-II). Study 3 •α =0.94 •Validity: Replication of mediation model with life event stressors (ASLEC) as covariate. TCAQ (but not SRRS) had a mediating role between neuroticism (EPQ-N) and depressive symptoms (SDS).
Figueira et al. (2017); Brazil	TCAQ-25 Portuguese-Brazilian	Experimental study	Undergraduate students	26	22.2 (5.5) 69.2%	•Validity: During the induced unpleasant emotional state, lower thought control ability (TCAQ) was related to lower working memory (WM, *r* = −0.57) capacity. This correlation was not significant for the neutral emotional state. Thought control is understood as the capacity to rapidly update information in WM based on demand (cognitive flexibility). Those scoring high on the TCAQ are able to maintain goal-relevant information over time and to shield goal-relevant information from intrusive information in order to adapt behavior to changing demands.

*ASLEC, Adolescent Self-Rating Life Events Checklist; BAI, Beck Anxiety Inventory; BD, Bipolar disorder; BDI-I/BDI-II, Beck Depression Inventory; CFA, Confirmatory Factor Analysis; DASS-21, Depression Anxiety Stress Scale-21; EPQ, Eysenck Personality Questionnaire (N-Scale); EFA, Exploratory Factor Analysis; LOT, Life Orientation Test; MAAS, Mindful Attention Awareness Scale; MCSDS, Marlowe-Crowne Social Desirability Scale; MDD, Major Depressive Disorder; MOCI, Maudsley Obsessive–Compulsive Inventory; NEOPI-R, Personality Inventory-Revised (neuroticism); OCI-R, Obsessive Compulsive Inventory-Revised; PAI, Personality Assessment Inventory [SOM, Somatic Complaints; ANX, Anxiety; ARD, Anxiety-Related Disorders; DEP, Depression; MAN, Mania; PA, Paranoia; SCZ, Schizophrenia; DRG, Drug Problems; ALC, Alcohol Problems; BOR, Borderline Personality Disorder; ANT, Antisocial Personality Disorder; SUI, Suicidal Ideation; STR, Stress; NON, Non-support; RXR, Treatment Rejection]; PANAS, Positive and Negative Affect Schedule; PCA, Principal Component Analysis; PSWQ, Penn State Worry Questionnaire; PTSD, Posttraumatic Stress Disorder; RRS-RSQ, Ruminative Response Scale of the Response Styles Questionnaire; SC-35, Scale of guilt feelings; SCL-PTSD, Symptom Checklist-90-Revised Crime Related PTSD Scale; SCS-R, Social Connectedness Scale-Revised; SDS, Self-rating Depression Scale; SRRS, Short Ruminative Responses Scale; STAI-T, State-Trait Anxiety Inventory (trait version); TCQ, Thought Control Questionnaire; TLEQ, Traumatic Life Events Questionnaire; UPPS, Impulsive Behavior Scale; TSI, Thought Suppression Inventory—Revised; WBSI, White Bear Suppression Inventory. N.A, Data not available*.

Most studies (10/17) examined undergraduate students and five studies investigated exclusively healthy adults (Gootjes et al., 2011; Gootjes and Rassin, 2014; Küpper et al., 2014; Rodríguez-Martín et al., 2015; van Schie et al., 2016). Three studies reported data of patient populations (Catarino et al., 2015; Piguet et al., 2016;Lu et al., 2017—Study 3).

A considerable proportion of studies (*n* = 8; 47%) presented cross-sectional/correlational results, 7 papers reported results from experimental tasks, and 4 studies were psychometric reports (one of them, van Schie et al., 2016, was focused on the psychometric properties of the TSI but included the TCAQ for comparison).

The original 25-items version of the TCAQ was administered in a majority of the included studies, whereas six papers presented results from shortened versions (Gay et al., 2008, 2011; Williams et al., 2010; Valdez and Lilly, 2012; Rodríguez-Martín et al., 2015; Piguet et al., 2016; Strauss et al., 2016; TCAQ-23 or TCAQ-20).

Regarding the mode of administration, most of the studies in the present review included the TCAQ in written form (paper-and-pencil version), while four studies used a computer-administered version of the instrument (Luciano et al., 2005; Luciano and Algarabel, 2006; Peterson et al., 2009;Valdez and Lilly, 2012).

### Quality Assessment of the Psychometric Properties

Table 3 presents the methodological quality per each measurement property of the TCAQ. The quality rating achieved was eleven out of a possible fourteen, indicating that overall the TCAQ measures thought control ability with reasonable levels of reliability and validity.

**Table 3:** Quality rating of the psychometric properties of the TCAQ and studies addressing (directly or indirectly) each psychometric domain.

**Content validity**	**Factor structure**	**Internal consistency**	**Test retest reliability**	**Validity**	**Floor/ceiling effects**	**Interpretability**	**Total/14**
2	2	2	1	2	0	2	11
Luciano et al. (2005) Gay et al. (2008)	Luciano et al., 2005; Gay et al., 2008; Williams et al., 2010	Luciano et al., 2005; Gay et al., 2008, 2011; Grisham and Williams, 2009; Peterson et al., 2009; Williams et al., 2010; Valdez and Lilly, 2012; Strauss et al., 2016; van Schie et al., 2016; Lu et al., 2017	Luciano et al., 2005; Williams et al., 2010	Luciano et al., 2005; Gay et al., 2008, 2011; Grisham and Williams, 2009; Peterson et al., 2009; Williams et al., 2010; Valdez and Lilly, 2012; Gootjes and Rassin, 2014; Küpper et al., 2014; Catarino et al., 2015; van Schie et al., 2016; Figueira et al., 2017; Lu et al., 2017	Not reported	Luciano et al., 2006; Gay et al., 2008; Peterson et al., 2009; Williams et al., 2010; Küpper et al., 2014; Catarino et al., 2015; Rodríguez-Martín et al., 2015; Piguet et al., 2016	

*Rating: 0, criterion not met/insufficient data to rate criterion; 1, criterion partially met; 2, criterion fully met. Due to the heterogeneity of the included studies, the methodological quality of each single study was not evaluated. The aim of several of the included studies was not the direct evaluation of one or more measurement properties of the TCAQ, but they provided indirect evidence of its validity [for instance, van Schie et al., 2016 used the TCAQ in a validation study of the Thought Suppression Invent ory—Revised*.

#### Content Validity

This criterion was considered satisfactory (2 points) because items were selected from other validated instruments (mainly WBSI and TCQ) and new items were generated after brain storming and consensus discussions within the research team (Luciano et al., 2005), who were experts on the field of thought suppression/thought control. The final version of the questionnaire comprised 25 items related to the perceived ability to control unwanted, intrusive thoughts. This version has been the most used worldwide (in 10 out of 16 studies). Shorter versions of the TCAQ have been developed (there is one with only 7 items; Rodríguez-Martín et al., 2015). For instance, according to Gay et al. (2008), the TCAQ contains two items (item 5 – “*I constantly censure my thoughts and actions*” and item 8 – “*I constantly evaluate whether my thoughts and actions are appropriate*”) specifically measuring behavior control instead of thought control, and for this reason, they decided to erase them when developing the French version. This 23-item TCAQ has been used in subsequent studies (Gay et al., 2011; Piguet et al., 2016) demonstrating good psychometric properties. The English version used by Williams et al. (2010), is shorter given removal of five items with low factor loadings after computing a CFA (items 5 and 8 -reported above- *plus* item 7 – “*I am usually successful when I decide not to think about something*,” item 14 – “*There are few things in life that manage to trouble me*,” and item 25 – “*I have much patience, and I do not lose my composure easily*”). To date, the accumulated empirical evidence supports the use of the TCAQ-20 in undergraduate students (Williams et al., 2010; Valdez and Lilly, 2012).

*Factor structure*. Dimensionality analyses have been performed in three studies (Luciano et al., 2005; Gay et al., 2008; Williams et al., 2010) to determine if the TCAQ items form one underlying dimension or multiple components. Using an exploratory approach, Luciano et al. (2005) and Gay et al. (2008) provided moderate evidence for a one-factor structure taking Kaiser's criterion, scree plot, and factor loadings into consideration. Williams et al. (2010) CFA of both the TCAQ-25 and TCAQ-20, yielded an acceptable level of support for the one factor model. We assigned 2 points to the TCAQ structural validity.

#### Internal Consistency

Once we had established that the unidimensionality of the TCAQ was clear, the next step was to check the interrelatedness of items. We found strong evidence (2 points) for solid internal consistency in the general factor measured in the TCAQ. Reliability has been examined through Cronbach's α in eight studies (Luciano et al., 2005; Grisham and Williams, 2009; Peterson et al., 2009; Williams et al., 2010; Gay et al., 2011; Valdez and Lilly, 2012; Rodríguez-Martín et al., 2015; Lu et al., 2017) and with an equivalent index in one study (Gay et al., 2008). Alpha values were around 0.90 across samples from all different countries (Spain, USA, China, etc.).

#### Test–Retest Reliability

Temporal stability was assessed in only two studies (Luciano et al., 2005; Williams et al., 2010), where circumstances were assumed to have remained stable over time. Test-retest reliability of the TCAQ has been excellent using Pearson correlations 0.68 (6 months; Williams et al., 2010) and 0.88 (2 months; Luciano et al., 2005). However, neither study reported the ICC for assessing stability, therefore this psychometric domain was scored with 1 point.

#### Convergent/Divergent Validity

Undoubtedly, this is the most explored psychometric domain (12 studies) in the TCAQ. Convergent validity was supported when examined by Pearson correlations between the TCAQ score and other self-reported measures. Thus, the reported coefficients were in the expected directions, being either moderate in most cases or strong in others (trait anxiety and neuroticism). Given that several correlations with related measures were ≥0.50, we scored this psychometric domain with 2 points. Of clinical relevance it is the utility and predictive capacity of the TCAQ in experimental tasks. In a standard thought suppression experiment, Grisham and Williams (2009) found that self-rated thought control ability was a significant predictor of frequency of a negative target thought. This result was further replicated in a subsequent experiment by Williams et al. (2010), who reported that low TCAQ scores were associated with higher frequency, distress, and suppression efforts when subjects were instructed to suppress a self-relevant thought. In a think/no-think task, Küpper et al. (2014) found that healthy adults scoring high in the TCAQ had more inhibitory control (voluntary forgetting) than participants with a low perceived control. The same research group replicated the experiment comparing adults with PTSD and trauma-exposed adults without PTSD (Catarino et al., 2015). Beyond the presence of PTSD and after controlling for depressive symptoms, TCAQ scores predicted suppression-induced forgetting. Finally, the link between thought control ability (as measured with the TCAQ) and working memory capacity was recently established using an electrophysiological index (Figueira et al., 2017). Thus, regardless of whether studies employed subjective (self-report measures) or objective measures (experimental tasks), convergent validity of the TCAQ is strongly supported.

#### Floor and Ceiling Effects

None of the studies provided information for floor or ceiling effects. For this reason, this domain was scored with 0 points.

#### Interpretability

Subgroup analyses have been undertaken in 50% of the studies (see Table 3), showing that, for example, *repressors* (low anxiety + high social desirability), and *low anxious* (low anxiety + low social desirability) individuals reported significantly higher TCAQ scores than did *high anxious* (high anxiety + low social desirability) and *defensive high anxious* (high anxiety + high social desirability) individuals (Luciano et al., 2006). In terms of gender and age, men obtained significantly higher TCAQ scores than women (Gay et al., 2008; Peterson et al., 2009; Williams et al., 2010) and older participants have scored significantly higher compared to younger participants (Gay et al., 2008). Rodríguez-Martín et al. (2015) found that individuals who were overweight reported less though control ability than normal-weight participants. In terms of clinical interpretability, Piguet et al. (2016) provided mean TCAQ scores for patients with a mood disorder (major depression or bipolar disorder) and healthy adults, reporting lower scores in the clinical samples relative to healthy controls. Overall, this domain was scored with 2 points.

## Discussion

In the last two decades, perceived thought control has been considered a higher-order construct in models of psychopathology, with research indicating it's relevance as a transdiagnostic predictor of clinical symptoms across mood and anxiety disorders (Brown and Barlow, 2009) and a predictor of treatment outcomes (Norton and Paulus, 2016). The construct has primarily been captured via self-report measures, such as the TCAQ. The main aim of the present work was to review the published results regarding the use and psychometric performance of the TCAQ across different areas of research and cultural contexts. We conducted a systematic search following PRISMA guidelines and adopted an up-to-date methodology (adapted COSMIN approach) to assess quality. Of 167 search hits, 17 papers met inclusion criteria and provided data on several psychometric indices.

The review indicates that the TCAQ has been used in a variety of populations, with samples of both healthy individuals and patients, including adults with PTSD (Catarino et al., 2015), and patients with major depression or bipolar disorder (Piguet et al., 2016; Lu et al., 2017). Nevertheless, more investigations with the TCAQ involving clinical samples are clearly warranted given 14 out of 17 studies (82.4%) employed undergraduate students.

Three studies have evaluated the dimensionality of the TCAQ (Luciano et al., 2005; Gay et al., 2008; Williams et al., 2010), supporting the unidimensionality of the measure. However, debate remains about the number of items that should be retained. Like in the case of the TCQ (Luciano et al., 2006), the WBSI (Schmidt et al., 2009), and TSI (Rassin, 2003), further questionnaire revision and item refinement seems warranted. Gay et al. (2008) proposed removal of Items 5 and 8 because they capture behavior control. Williams et al. (2010) eliminated five items (5, 7, 8, 14, and 25) with low standardized factor loadings. Therefore, both groups of research coincide to eliminate items 5 and 8. Additional CFAs in larger samples of healthy individuals and clinical samples are necessary to confirm these findings.

An important issue of the present review relates to the validity of the TCAQ, which can be defined as the degree to which the instrument actually measures what it intends to measure. Overall, this review supports contemporary theories that conceive low perceived control as a general psychological vulnerability factor involved in the etiology and maintenance of emotional disorders (Gallagher et al., 2014) as well as experimental research (*think/no-think paradigm*; for a resent review see Engen and Anderson, 2018) indicating that deficits in control ability and inhibition represent an important vulnerability factor for psychiatric disorders such as PTSD (Catarino et al., 2015). Taking a dimensional approach of psychopathology as framework, Piguet et al. (2016) proposed thought control deficits as a common vulnerability trait that surpasses the diagnostic boundaries, which aligns well with the NIMH Research Domain Criteria (RDoC) initiative (Sanislow et al., 2010). Thus, we identified studies reporting correlations between the TCAQ and many relevant measures of psychopathology (e.g., anxiety, depression, worry, neuroticism) (e.g., Gay et al., 2008; Peterson et al., 2009; Gootjes and Rassin, 2014; Lu et al., 2017). In line with this, Benight and Bandura (2004) posited that “*thought control self-efficacy*” is one of the four main cognitive mechanisms by which self-efficacy promotes emotional well-being. According to these authors, people who can effectively manage unwanted thoughts and not ruminate about them are better at regulating their emotional states. Moreover, how well-individuals can stop themselves from ruminating about undesirable things can help them to focus on present activities (i.e., exert attentional control). Interestingly, when Gootjes and Rassin (2014) analyzed the link of mindfulness, perceived thought control ability (TCAQ), and psychological functioning (trait anxiety and negative affect) using a mediation model (path analysis), these authors found that TCAQ scores fully mediated the relationship between hours spent meditating and trait anxiety as well as the relation between hours spent meditating and negative affect. Furthermore, the authors reported that TCAQ scores also fully mediated the relationship between hours spent meditating and variables related to healthy psychological functioning (e.g., positive affect, dispositional optimism, and social connectedness). Given the burgeoning application of mindfulness and meditation practice (see Goldberg et al., 2018 for recent review), this potential link warrants further study and highlights the need for a validated measure of thought control ability to assess the nature of the relationship (i.e., uni- vs. bidirectional).

Based on the summary findings, the TCAQ appears to be a unidimensional, reliable, and valid instrument for use in quantitative research. To date, TCAQ psychometric analyses have employed classic test theory as a framework. This approach does not permit assessment of the quality of individual TCAQ items and response options across different levels of thought control. The use of methods based on item response theory would provide specific information about the functioning of each TCAQ item, as has already been done with the WBSI (Palm and Strong, 2007; Schmidt et al., 2009), and would allow assessment of differential item functioning. Although the measure has been used in samples from diverse cultures and with different languages, few feasibility analyses have been conducted. Aspects such as time needed for completion, missing data related to difficulties in understanding the items, or the measure's acceptability have not been explored. To our knowledge, the included studies did not report the percentage of missing items or describe how missing items were managed, which may have introduced bias in the findings, and therefore lowered the study quality. There is also lack of data regarding measurement invariance. No multiple group factor analysis has been performed. Moreover, as only a few of the included studies had a longitudinal design, we are not able draw firm conclusions about the temporal stability of the TCAQ. Further, some studies employing small sample sizes (<30 participants) were included here (Gootjes et al., 2011; Figueira et al., 2017), so they may have been underpowered and their results should be interpreted with caution. Consequently, continued psychometric evaluation in larger populations with a longitudinal design seems warranted.

## Conclusions

To our knowledge, the present systematic review is the first to summarize the use of the TCAQ in different research fields. The TCAQ has been included in studies of a variety of populations, in samples of healthy subjects and patients. We applied adapted COSMIN criteria to evaluate the quality of the TCAQ measurement properties; and provided a comprehensive and qualitative synthesis of its current evidence. The quality rating achieved was 11 out of a possible 14. Overall, the dimensionality, reliability and construct validity assessed was shown to be adequate. Overall, considering the accumulated empirical data, we recommend the use of the TCAQ as a tool to assess perceived control of unwanted thoughts. The current systematic review supports the inclusion of thought control ability in CBT models of mood and anxiety disorders, and provides further support for the TCAQ as an empirically-validated tool that has incremental value in the prediction of psychopathological symptoms over other existing thought control-related self-report measures (WBSI, TCQ, and TSI). Nevertheless, future clinical studies are needed to delineate how perceived thought control ability and other thought control-related constructs (such as thought suppression, thought control strategies etc.) interact with each other in order to determine their unique and potentially interactive role in the prediction of psychopathological symptoms.

## Author Contributions

AP-A and JL made substantial contribution to the analysis and to the interpretation of the data, drafted the manuscript, provided final approval of the version to be published, and agreed to be accountable for all aspects of the work in ensuring that questions related to the accuracy or integrity of any part of the work are appropriately investigated and resolved. AF-S, JM-M, LA-R, NA-O, PH-M, and AW helped out in the interpretation of data for the work, revised the manuscript critically for important intellectual content, provided final approval of the version to be published, and agreed to be accountable for all aspects of the work in ensuring that questions related to the accuracy or integrity of any part of the work are appropriately investigated and resolved.

### Conflict of Interest Statement

The authors declare that the research was conducted in the absence of any commercial or financial relationships that could be construed as a potential conflict of interest.
