# Supplementary material for: Fifteen Years Controlling Unwanted Thoughts: A Systematic Review of the Thought Control Ability Questionnaire (TCAQ)
Source: Front Psychol. 2019 Jun 19;10:1446. doi: 10.3389/fpsyg.2019.01446 (PMC6593181; doi:10.3389/fpsyg.2019.01446)
Supplement: Supplementary file 1 [file Table_1.DOC]

**Supplementary Table 1**. Item content of the TCAQ

|  | **A** | **B** | **C** | **D** | **E** |
| --- | --- | --- | --- | --- | --- |
| **1.** It is often difficult for me to fall asleep because my mind keeps going over personal problems^a^ |  |  |  |  |  |
| **2.** I often cannot avoid having upsetting thoughts^a^ |  |  |  |  |  |
| **3.** Although some people criticize me unfairly, I can’t help thinking they might be right^a^ |  |  |  |  |  |
| **4.** I manage to have control over my thoughts even when under stress |  |  |  |  |  |
| **5.** I constantly censure my thoughts and actions^a,b^ |  |  |  |  |  |
| **6.** Any setback overwhelms me, no matter how small^a^ |  |  |  |  |  |
| **7.** I am usually successful when I decide not to think about something^b^ |  |  |  |  |  |
| **8.** I constantly evaluate whether my thoughts and actions are appropriate^a,b^ |  |  |  |  |  |
| **9.** It is very easy for me to stop having certain thoughts |  |  |  |  |  |
| **10.** I feel worried, frustrated or sad for a long time after having an embarrassing, troublesome or painful experience^a^ |  |  |  |  |  |
| **11.** It is easy for me to free myself of troublesome thoughts |  |  |  |  |  |
| **12.** Frequently, some thoughts or images take over my mind^a^ |  |  |  |  |  |
| **13.** There are negative things in my past that I cannot help remembering^a^ |  |  |  |  |  |
| **14.** There are few things in life that manage to trouble me^b^ |  |  |  |  |  |
| **15.** I haven’t been able to get the argument I had with (my partner, my parents, a friend…) out of my head for several days^a^ |  |  |  |  |  |
| **16.** I consider myself a person who is good at controlling positive and negative emotions |  |  |  |  |  |
| **17.** My thoughts control me more than I control them^a^ |  |  |  |  |  |
| **18.** There are some thoughts that enter my head without me being able to avoid it^a^ |  |  |  |  |  |
| **19.** My thoughts are uncontrollable^a^ |  |  |  |  |  |
| **20.** I am not usually overwhelmed by unpleasant thoughts |  |  |  |  |  |
| **21.** I am unable to free myself from certain thoughts: e.g. “I am a failure”, “I am useless”, “I am no good at all”, etc^a^ |  |  |  |  |  |
| **22.** I think other people have more control over their thoughts than I do^a^ |  |  |  |  |  |
| **23.** If I get angry or fight with someone, I can’t stop thinking about it, and I can hardly work or concentrate^a^ |  |  |  |  |  |
| **24.** I get rid of uncomfortable thoughts or images almost effortlessly |  |  |  |  |  |
| **25.** I have much patience, and I do not lose my composure easily^b^ |  |  |  |  |  |

**Scoring:**

A=1; B=2; C=3; D=4; E=5

^a^ Reverse scored items;

^b^ Item removed from the 20-item version
